# Supplementary material for: Dynamic modulation of the lipid translocation groove generates a conductive ion channel in Ca2+-bound nhTMEM16
Source: Nat Commun. 2019 Oct 31;10:4972. doi: 10.1038/s41467-019-12865-4 (PMC6823365; doi:10.1038/s41467-019-12865-4)
Supplement: Supplementary file 1 — Supplementary Information [file 41467_2019_12865_MOESM1_ESM.pdf]

SUPPLEMENTARY INFORMATION

Dynamic modulation of the lipid translocation groove generates a conductive ion channel in Ca^{2+}-bound nhTMEM16

George Khelashvili, Maria E Falzone, Xiaolu Cheng, Byoung-Cheol Lee,

Alessio Accardi, Harel Weinstein

SUPPLEMENTARY NOTE 1

The gating mechanism from the large-scale MD simulations

For the detailed analysis of conformational dynamics of the groove region of the protein from the MD trajectories we utilized the same tICA approach, with the same set of collective variables (CVs) as described before [1] (see Methods). These CVs describe the dynamics of the polar network of residues comprising the E313/E318/R432 triad that forms the extracellular gate, and the T333/Y439 pair that forms the mid-point constriction of the lipid pathway. Thus, the list of residue-pair distances used as the collective variables in the dimensionality reduction analysis with the tICA approach (see Methods and Supplementary Table 2) is composed of the following: T333-Y439; E313-R432; E318-R432; Y439-R432; and V337-V447. To this group of CVs we added another dynamic parameter, the V337-V447 distance, which informs on the distance between the central regions of helices TM4 and TM6 that were seen to rearrange in the simulations. The tICA representation of the enhanced complement of trajectories sampling more of the rare events, in the space of the larger group of CVs, was still found to contain the same microstate regions and transitions identified previously to describe lipid permeation [1]. Figure 1A shows the projection of the full dataset of trajectories on the 2D space of the first two vectors of the tICA analysis (tIC_{WT} 1 and tIC_{WT} 2) which, as shown in Supplementary Figure 1A-B, describe ~80% of the total dynamics of the system.

The discretization of the 2D tICA space from Figure 1A into 50 microstates (Supplementary Figure 1A) led to identification of those regions on the tICA landscape that represent the complete lipid flipping. As shown in Supplementary Figure 2, as the lipid is translocated through the groove from the IC to EC direction, the system evolves from the crystal structure-like conformations (microstate 1) to microstates in which the EC gates are partially, or fully disrupted (microstates 6 and 5, respectively). Microstate 6 also illustrates an incomplete flip mechanism in which the lipid remains coordinated by the protein residues at the extracellular end of the groove. In this case, the T333-Y439 and E318-R432 interactions are broken, but the E313-R432 interaction is still intact and hence not all flipping conditions are met (see structural representations in Supplementary Figure 2H). Indeed, as shown previously, all three gating interactions need to be disrupted for the lipid to be released from the groove to the EC leaflet [1,2]. States with all three gates open are predominant in microstate 5 (Supplementary Figure 1D). Thus, the mechanism emerging from the tICA analysis from the extensive sampling provided by the trajectories analyzed here, recapitulates the gating dynamics of the Ca^{2+}-bound nhTMEM16 reported in our previous studies [1].

However, in the larger set of trajectories the previously observed “rare events” are sampled in a sufficient number to enable detailed analysis of the structural underpinnings.

Supplementary Figure 1

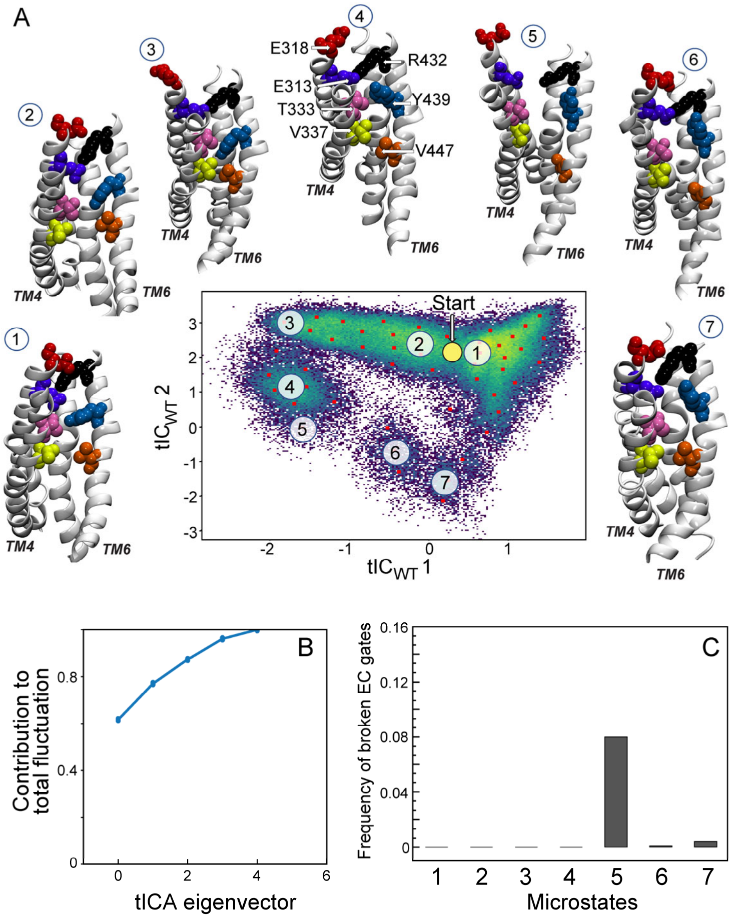

Supplementary Figure 1: Structural characteristics of states in the lipid translocation mechanism. (A) Central panel:2-D landscape representing all the trajectories of the WT nhTMEM16 protein (Supplementary Table 1) mapped with the tICA transformation in the space of the first two tICA eigenvectors (tIC_{WT} 1 and tIC_{WT} 2, see Methods for details of tICA analysis). The lighter shades (light green to yellow) indicate the most populated regions of the 2D space. The dynamics of the two subunits of the protein in each trajectory were considered separately in the analysis. Microstates (see Methods) representing the most populated states in these simulations are indicated by the numbered circles and represent various stages in the lipid translocation process. Representative structures of each microstate are shown in the surrounding snapshots. In these structural models, the relevant groove residues appear in space fill representations and are labeled in microstate 4, TM4 and TM6 helices are labeled. The location of the initial conformation of the system (the 4WIS X-ray model) is indicated on the 2D tICA landscape by the yellow circle marked "Start". (B) Contribution of each tIC_{WT} vector to the total fluctuation of the WT nhTMEM16 system. (C) For each microstate in panel A the plot compares the fractions of trajectory frames in the respective microstate in which the three functional EC gates, T333-Y439, E313-R432, and E318-R432, are simultaneously broken (the T333-Y439 gate was assumed to be broken if the T333-Y439 distance was > 8Å; the E313-R432 and E318-R432 gates were assumed to be broken if the distance between carbonyl oxygen of Glu and sidechain nitrogen of Arg was > 6Å).

Supplementary Figure 2

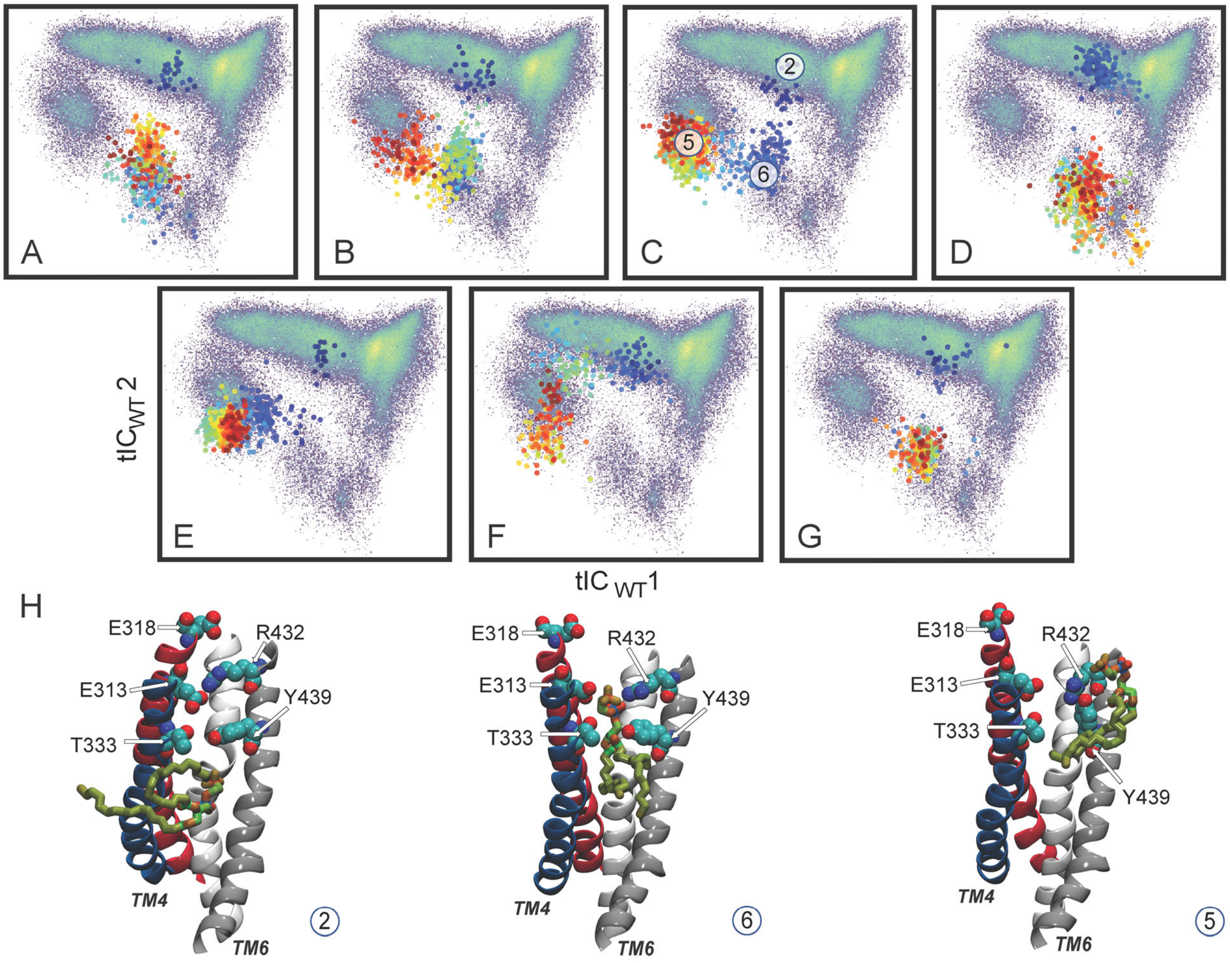

Supplementary Figure 2: Structural characteristics of various stages during the lipid flipping process in the WT nhTMEM16. (A-G) Projections of the seven trajectories from the $WT^{ensemble}$ simulations in which the lipid flipping from the IC to the EC leaflet was observed. The color code is the same as in Figure 2A. In panel C, locations of the Microstates 2, 6, and 5 are highlighted as in Figure 1A, and their structural representations are shown in panel H (see text for more details). (H) Structural representations of Microstates 2, 6, and 5. Relevant TM helices are drawn in cartoon and colored, TM4 and TM6 helices are labeled, the key residues are depicted in van der Waals and are labeled, and the flipped lipid is illustrated in licorice.

Supplementary Figure 3

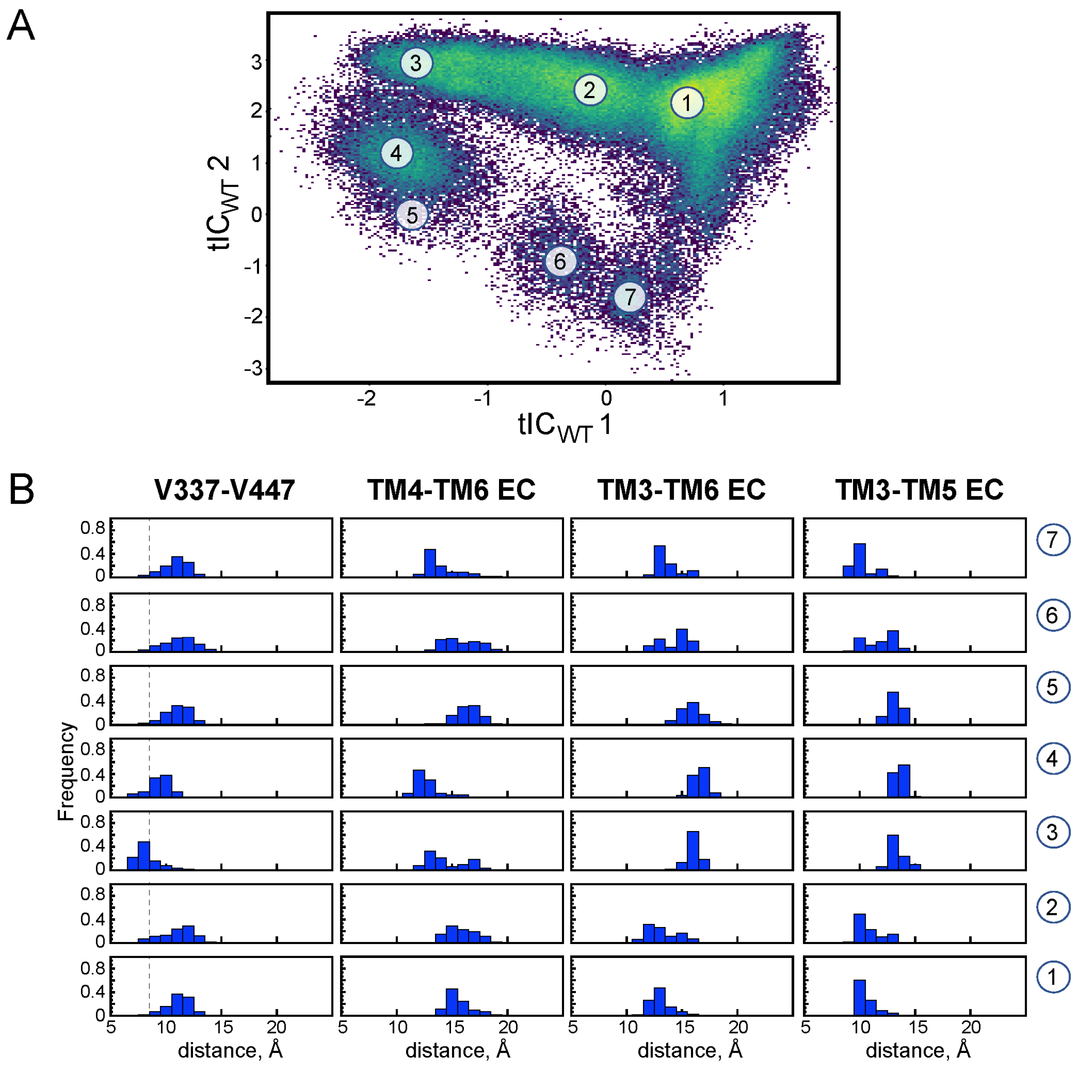

Supplementary Figure 3: Structural characteristics of various microstates obtained from tICA analysis of the WT nhTMEM16. (A) The 2D tICA landscape of the WT nhTMEM16 re-drawn from Figure 1A of the main text. (B) The columns from left to right record the probability distributions of the C_α-C_α distance between V337-V447 residues (the vertical dashed lines represent the 8.5Å distance cut-off used to define the occluded conformation of the groove), the C_α-C_α distance between F330-R432 residues (TM4-TM6 EC), distance between the center-of-mass of residue segments 315-318 and 432-435 (TM3-TM6 EC), and the C_α-C_α distance between E313-A385 residues (TM3-TM5 EC) in the selected 7 microstates from panel A.

Supplementary Figure 4

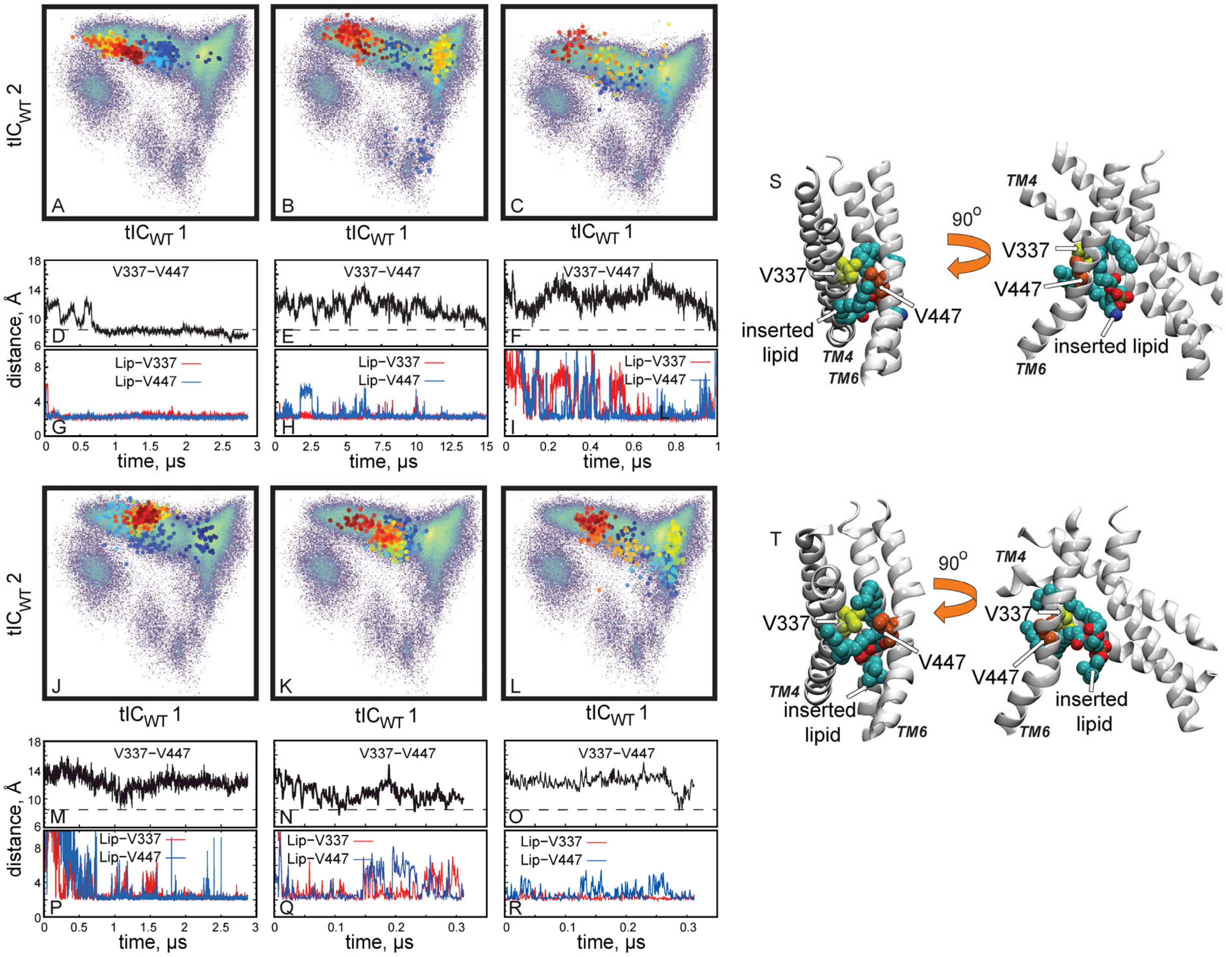

Supplementary Figure 4: Lipid tail insertion leads to the occlusion of the groove. (A-C and J-L) Projections on the 2D tICA landscape from Figure 1 of the wild type nhTMEM6 system from different trajectories in which the occlusion of the groove was observed – Panels A and J show data for the two subunits from the WT1 trajectory; Panel B shows results for one of the subunits from the WT6 trajectory (the same data for another subunit is shown in Figure 2); Panel C depicts the trajectory of one of the subunits from the WT4 simulation; and Panels K and L present data from two trajectories from the WT^{ensemble} set. Different time-frames of the trajectory are shown as colored dots with darker (blue, cyan) shades representing the initial stages of the simulation, lighter colors (yellow, green) corresponding to the middle part of the trajectory, and red shades showing the last third of the trajectory. (D-F and M-O) The C_α-C_α distance between V337 and V447 residues as a function of time in the trajectories shown in panels A-C and J-L, respectively. Horizontal dashed lines demarcate the 8.5Å distance cut-off used to define the occluded conformation. (G-I and P-R) Time evolution of the minimum distance between the lipid tail penetrating the groove and V337 (red) and V447 (blue) residues in the trajectories shown in panels A-C and J-L, respectively.

Supplementary Figure 5

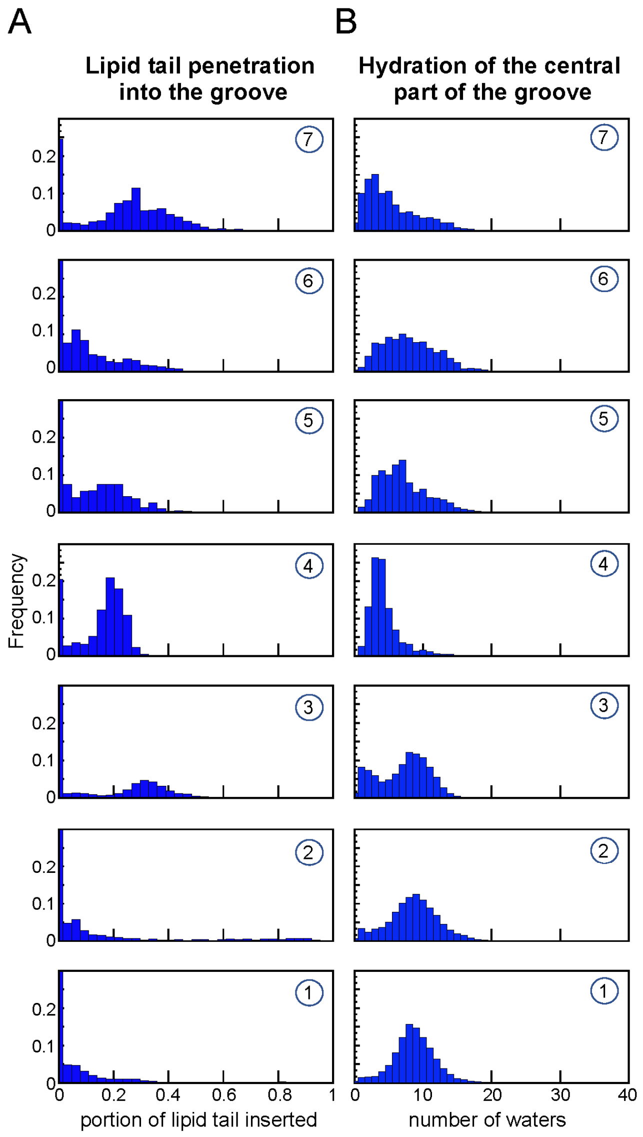

Supplementary Figure 5: Lipid tail penetration into the groove in the WT nhTMEM16 results in dehydration of the region. (A) The probability distributions of fraction of the lipid tail inserted in the groove in the 7 microstates defined in Figure 1A. To construct these histograms, the number of lipid tail atoms inserted into the groove was counted. A lipid tail atom was considered to lie in the groove if it was within 5Å of the sidechain of any of the following residues: 377, 378, 381, 382, 385, 501, 505, 509, and 513. The resulting atom count was then normalized by the total number of lipid tail atoms (which was 32 for all the lipids (POPE, POPG, and POPC) considered here). With this definition, a value of "1" corresponds to an entire lipid tail inserted into the groove. (B) The probability distributions of number of water molecules in the central region of the groove (defined as the groove area lined by the z-axis positions of the C_α atoms of residues T381 and Q436, see Figure 6; see also Methods) in the 7 microstates defined in Figure 1A.

Supplementary Figure 6

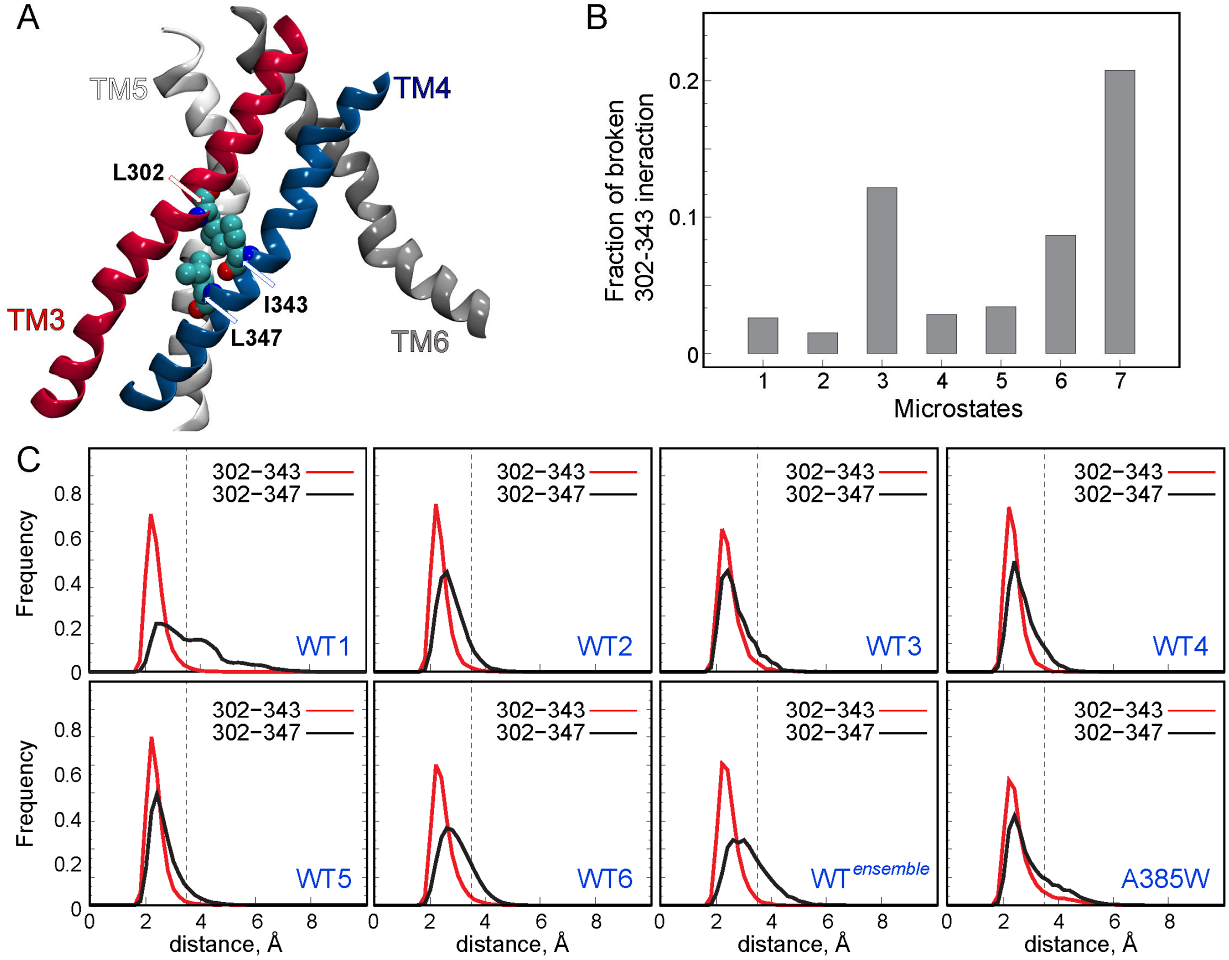

Supplementary Figure 6: TM3 and TM4 helices in nhTMEM16 are locked in the hydrophobic interactions mediated by L302/I343/L347 triad of residues. (A) Same as in Figure 3A of the main text – two views, related by 90° rotation around the membrane normal, of the wild type nhTMEM16 protein groove region showing locations of L302, I343 and L347 residues (in space fill and labeled). TM3, TM4, TM5, and TM6 helices lining the groove are depicted in red, blue, white and grey colors and labeled accordingly. (B) Fraction of trajectory frames in each Microstate from Figure 1A with the broken L302-I343 interaction. The interaction was assumed to be broken when the minimal distance between residues L302 and I343 was > 3.5Å. (C) Histograms of L302-I343 (red) and L302-L347 (black) distances are shown separately for all the simulations of the wild type nhTMEM16 and of the A385W system (see also Figure 3B and Supplementary Table 1). In each pane, a vertical dashed line demarcates the 3.5Å distance cut-off used to define a state in which the interactions between L302 and I343/L347 are broken.

Supplementary Figure 7

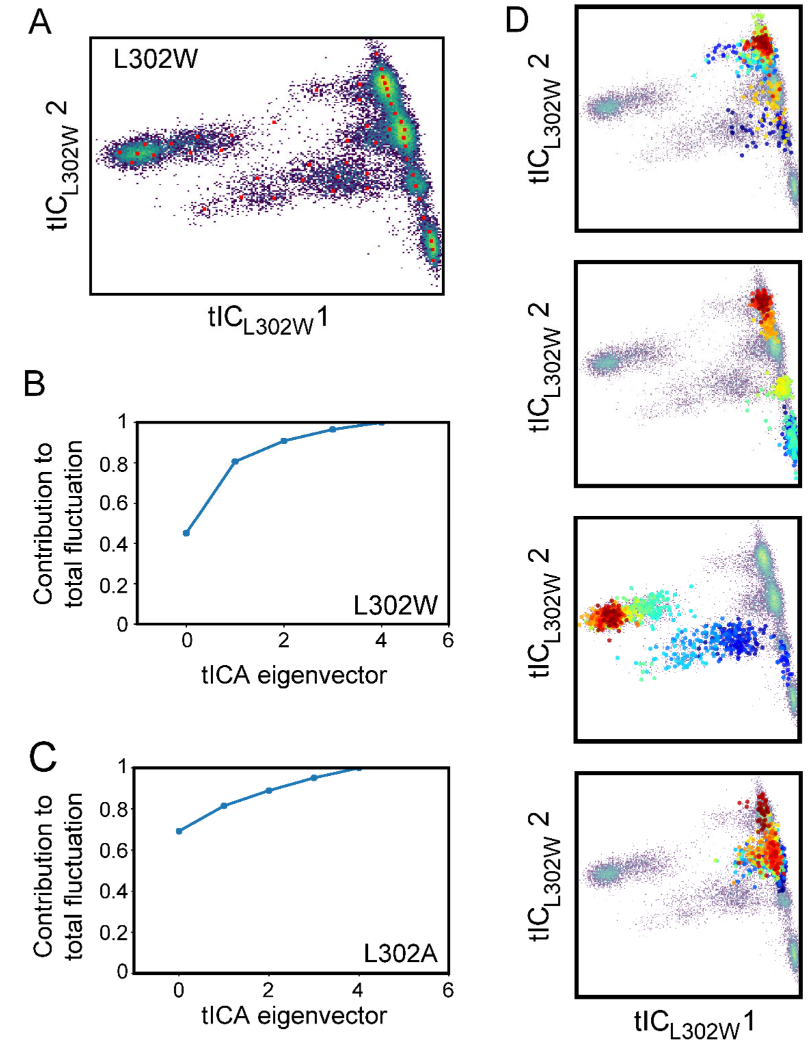

Supplementary Figure 7: Structural characteristics of various microstates obtained from tICA analysis of the L302 mutant systems. (A) The full set of the L302W nhTMEM16 trajectories mapped on the 2D landscape of the first two tICA eigenvectors (tIC$_{L302W}$ 1 and tIC$_{L302W}$ 2). Shown also are locations of the 50 microstates (red squares) obtained from the k-means clustering analysis of the conformational space. (B) Contribution of each tICA$_{L302W}$ vector to the total fluctuation of the L302W nhTMEM16 system. (C) Contribution of each tICA$_{L302A}$ vector to the total fluctuation of the L302A nhTMEM16 system. (D) Projection of the trajectories for the individual nhTMEM16 subunits from the L302W simulations (the top two and the bottom two panels correspond to the subunits of the same dimer).

Supplementary Figure 8

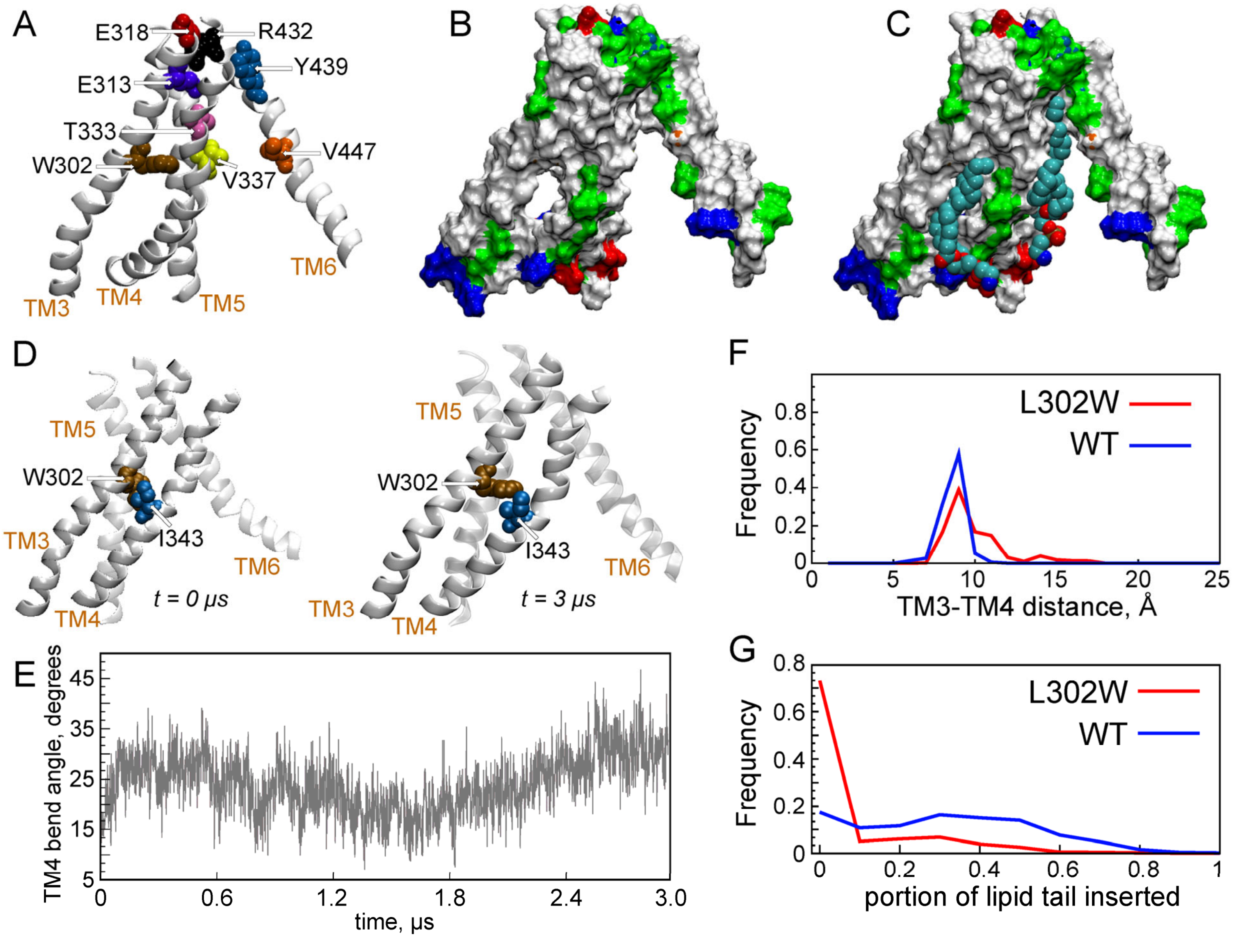

Supplementary Figure 8: Structural characteristics of various conformations of the groove in L302W nhTMEM16. (A) Snapshot from the L302W simulation illustrating large kink in TM4 helix. Relevant helices and residues are shown in cartoon and space fill representation, respectively, and labeled. (B) Surface rendering of the protein structure from panel A in which the protein residues are colored according to their type using the following color code: white – hydrophobic, green – polar, blue – basic, and red – acidic. Note large void between TM3 and TM4 helices surrounded by hydrophobic amino acids. (C) The view of the same structure as in B only showing lipids observed in the simulations to penetrate the groove region filling up the void shown in panel B. (D) Snapshots of the nhTMEM16 L302W at time-points t=0μs (initial) and t=3μs (final, same as in panel A) in one of the trajectories (see Supplementary Table 1), illustrating bending of helix TM4. Relevant helices are shown in cartoon, and residues space fill representation, and labeled. (E) Time-evolution of TM4 bend angle around the L302/I343 pair in the nhTMEM16 L302W trajectory from panel D. (F) Histogram of C_α-C_α distance between the residues at positions 302 and 343 for the wild type (blue) and L302W (red) system (i.e. L302-I343 for the WT and W302-I343 for the L302W). (G) Histograms of average fraction of lipid tail atoms inserted in the groove in the simulations of the wild type (blue) and L302W (red) nhTMEM16.

Supplementary Figure 9

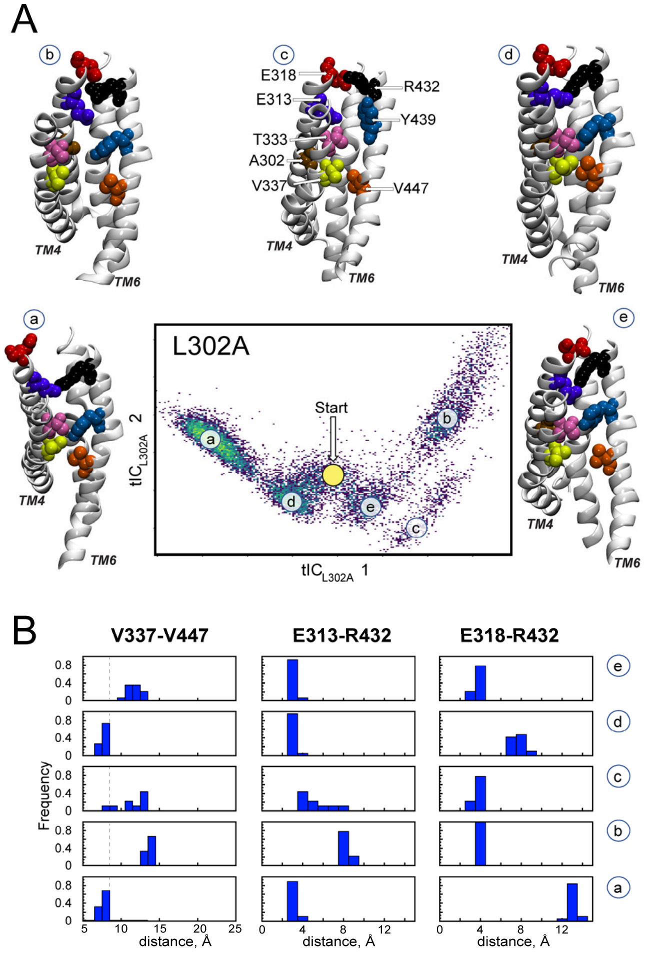

Supplementary Figure 9: Structural characteristics of various microstates obtained from tICA analysis of the L302A nhTMEM16 systems. (A) Central panel:2-D landscape representing all the trajectories of the L302A nhTMEM16 protein (Supplementary Table 1) mapped with the tICA transformation in the space of the first two tICA eigenvectors (tIC_{L302A} 1 and tIC_{L302A} 2, see Methods for details of tICA analysis). The lighter shades (light green to yellow) indicate the most populated regions of the 2D space. The dynamics of the two subunits of the protein in each trajectory were considered separately in the analysis. Microstates (see Methods) representing the most populated states in these simulations are indicated by the labeled circles ("a"-"e") and represent various stages in the lipid translocation process. The location of the initial conformation of the system (based on the 4WIS X-ray model) is indicated on the 2D tICA landscape by the yellow circle marked "Start". Representative structures of each microstate are shown in the surrounding snapshots. In these structural models, the relevant groove residues appear in space fill representations and are labeled in microstate "c", TM4 and TM6 helices are labeled. (B) The columns from left to right record the probability distributions of the C_α-C_α distance between V337-V447 residues (vertical dashed lines demarcate the 8.5Å distance cut-off used to define the occluded conformation of the groove), and of the distances between E313-R432 and E318-R432 pairs of residues (defined as the distance between carbonyl oxygen of Glu and sidechain nitrogen of Arg) in the selected 5 microstates from panel A.

Supplementary Figure 10

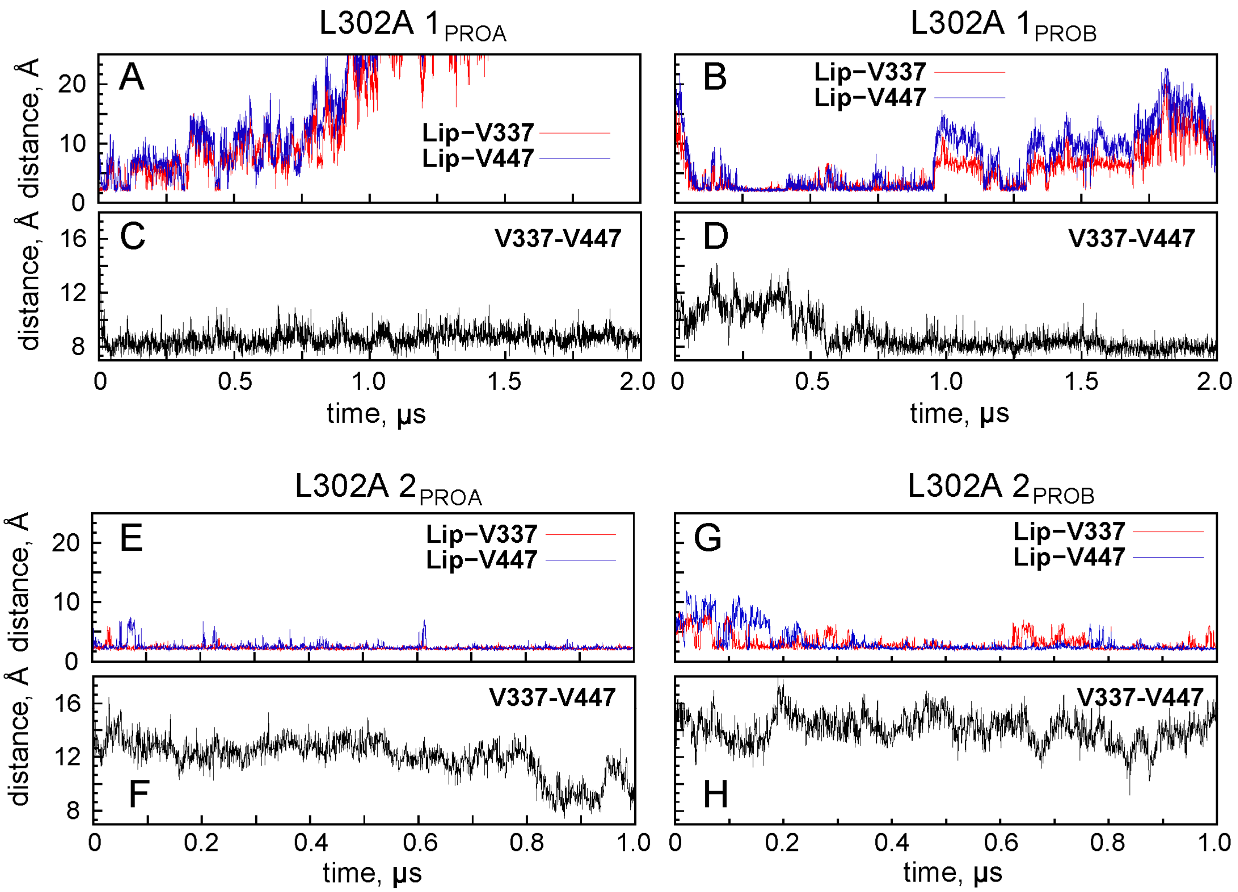

Supplementary Figure 10: Lipid tail insertion facilitates occlusion of the groove in the L302A nhTMEM16 system. Time-evolution of the minimum distance between the inserted lipid tail and V337 (red) and V447 (blue) residues (A) and of the C_α-C_α distance between V337-V447 residue pair (C) in one of the subunits of the L302A nhTMEM16 from microsecond-scale MD simulations. Panels B/D and E/F and G/H show the same quantities for the other subunits of the L302A mutant for which lipid penetration into the groove was observed.

Supplementary Figure 11

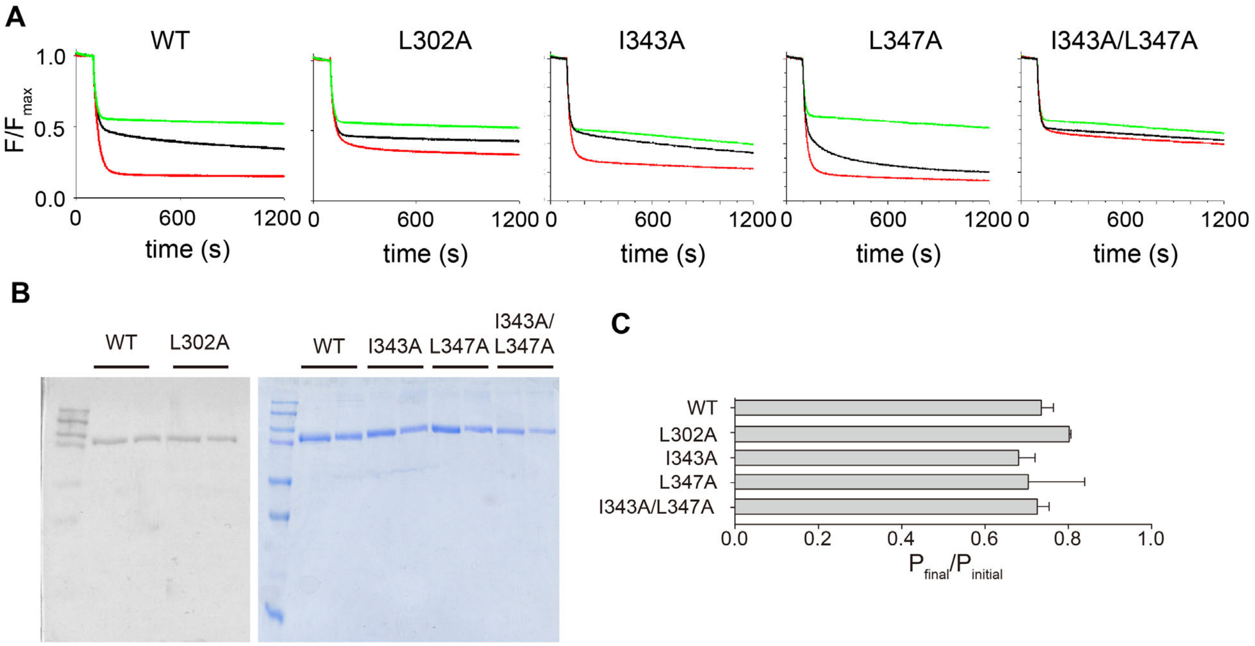

Supplementary Figure 11. Reconstitution of nhTMEM16 mutants in proteoliposomes for scrambling assay. A) Representative time courses of dithionite-induced fluorescence decay in protein-free liposomes (green) or in proteoliposomes containing WT or mutant (L302A, I343A, L347A or I343A/L347A) nhTMEM16 with (red) or without (black) Ca^{2+}. Dithionite was added at t=100 s. (B) SDS-PAGE gels of liposomes containing wt or mutant nhTMEM16 at the beginning (left lanes) and end (right lanes) of the reconstitution process confirming the presence of the protein. In all gels the final band represents the protein present in the liposomes when the experiment was completed. C) Quantification of the fraction of WT and mutant nhTMEM16 protein reconstituted in proteoliposomes. Bars represent the average from n=3+ independent reconstitutions. Error bar represents the S.E.M.

Supplementary Figure 12

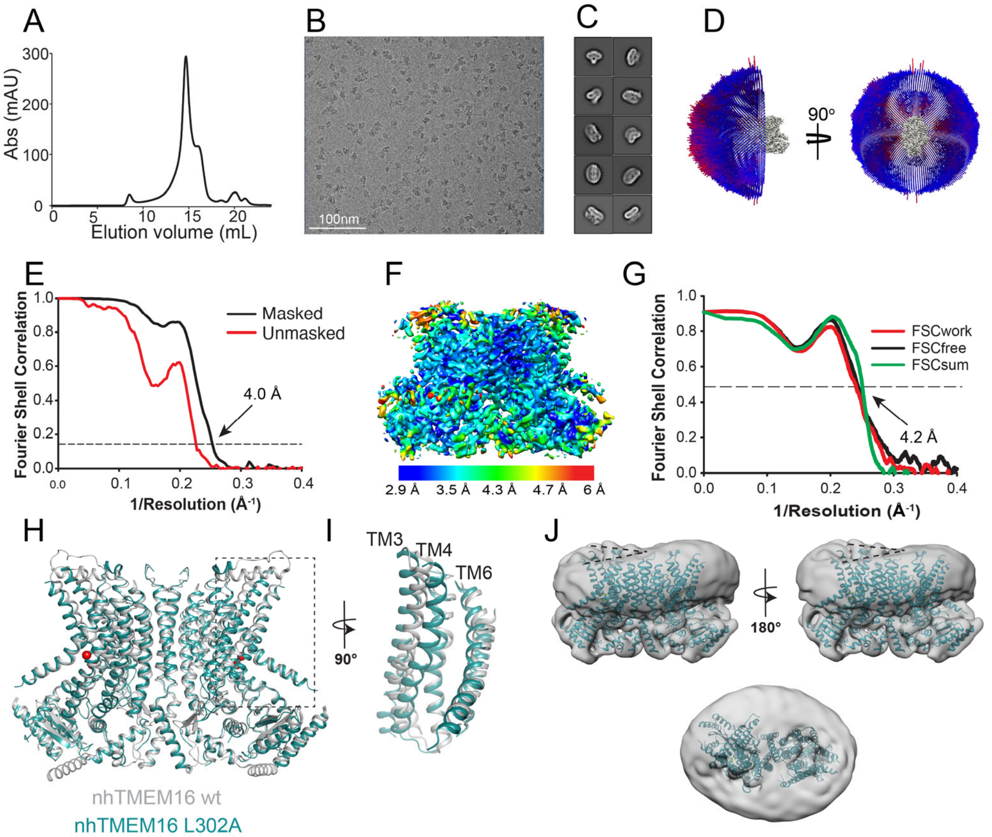

Supplementary Figure 12. Cryo-EM characterization of nhTMEM16 L302A reconstituted into nanodiscs. (A) Size exclusion profile of nhTMEM16 L302A in MSP1E3 nanodiscs. (B) Representative cryo-EM micrographs of vitrified nhTMEM16 L302A/nanodisc complex. (C) Representative 2D-class averages, box size 275 Å. (E) Angular distribution representation of final 3D reconstruction with C2 symmetry, number of views at each angular orientation is represented by length and color of cylinders where red indicates more views. (E) FSC plot indicating the resolution at the 0.143 threshold of final masked (black) and unmasked (red) map of nhTMEM16 L302A. (F) Final masked reconstruction colored by local resolution calculated using the Bsoft program BlocRes [3,4]. (G) FSC curves of refined models versus maps of nhTMEM16 L302A reconstituted in nanodiscs. Green: FSC curves for the refined model compared to the final masked reconstruction (FSCsum). Red: FSC curves for the modified, refined model compared to the masked half-map 1 (FSCwork, used during validation refinement). Black: FSC curves for the modified, refined model compared to the masked half- map 2 (FSCfree, not used during validation refinement). Dashed lines show FSC threshold used for FSCsum of 0.5. Statistics for the EM analysis and model building are reported in Supplementary Table 4. (H) Structural alignment of WT (4WIS, gray) and L302A nhTMEM16 (PBDID 6OY3, teal) shown in ribbon representation. Dashed lines denote permeation pathway. Ca^{2+} ions are shown as red spheres. (I) Structural alignment of the permeation pathway of WT (4WIS) and L302A nhTMEM16 (90° rotation from boxed region in H) (J) The unmasked map (gray) of the L302A nhTMEM16/nanodisc complex in the presence of 0.5 mM Ca2+ is shown low pass filtered to 10 Å at σ=0.4. The structural model of L302A nhTMEM16 (teal) is shown inside the unmasked map. Top panels: front (left) and back (right) view. Black dashed lines highlight the bending at the dimer cavities. Bottom panel: top view.

Supplementary Figure 13

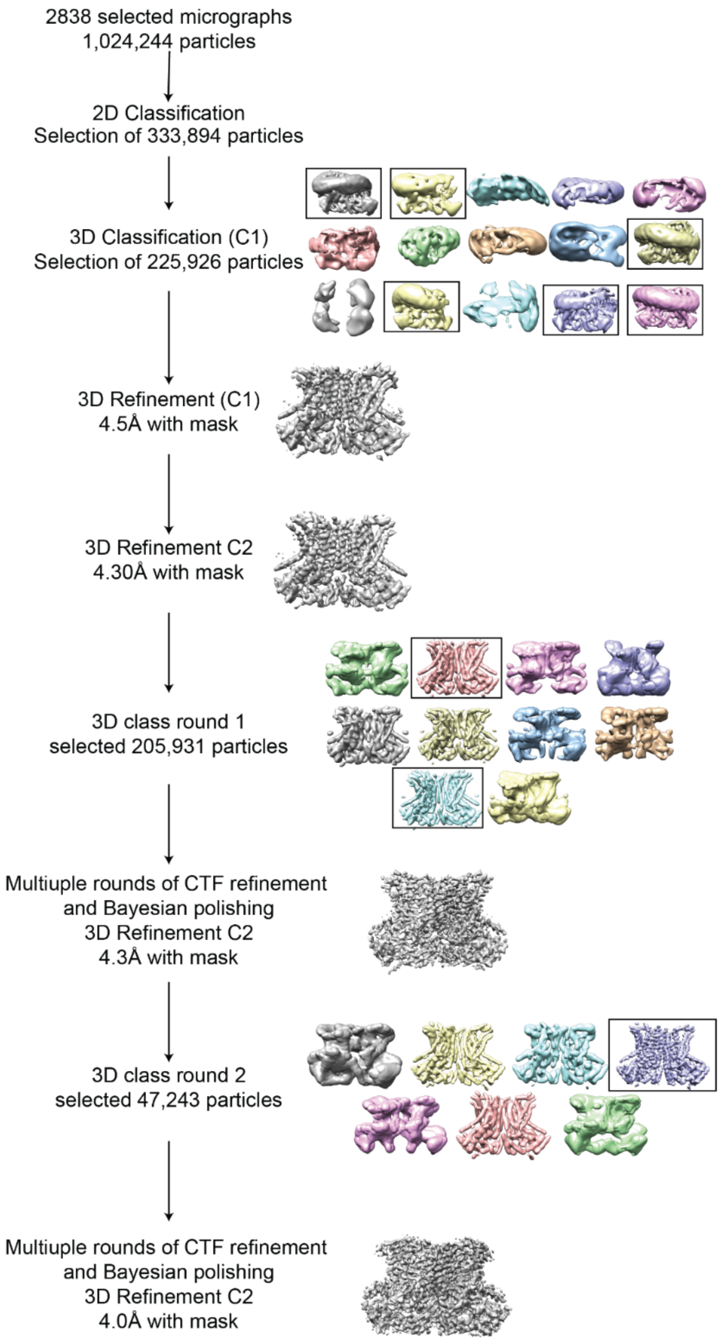

Supplementary Figure 13. Cryo-EM data processing procedure for the nhTMEM16 L302A/nanodisc complex. Particles picked from manually inspected micrographs were sorted with 2D and 3D classification in C1. Inspection of the first refinement revealed a two-fold symmetric arrangement so C2 symmetry was applied for further refinements and 3D classification. Several rounds of 3D classification, CTF refinement, and Bayesian polishing were carried out before the final masked refinement.

Supplementary Figure 14

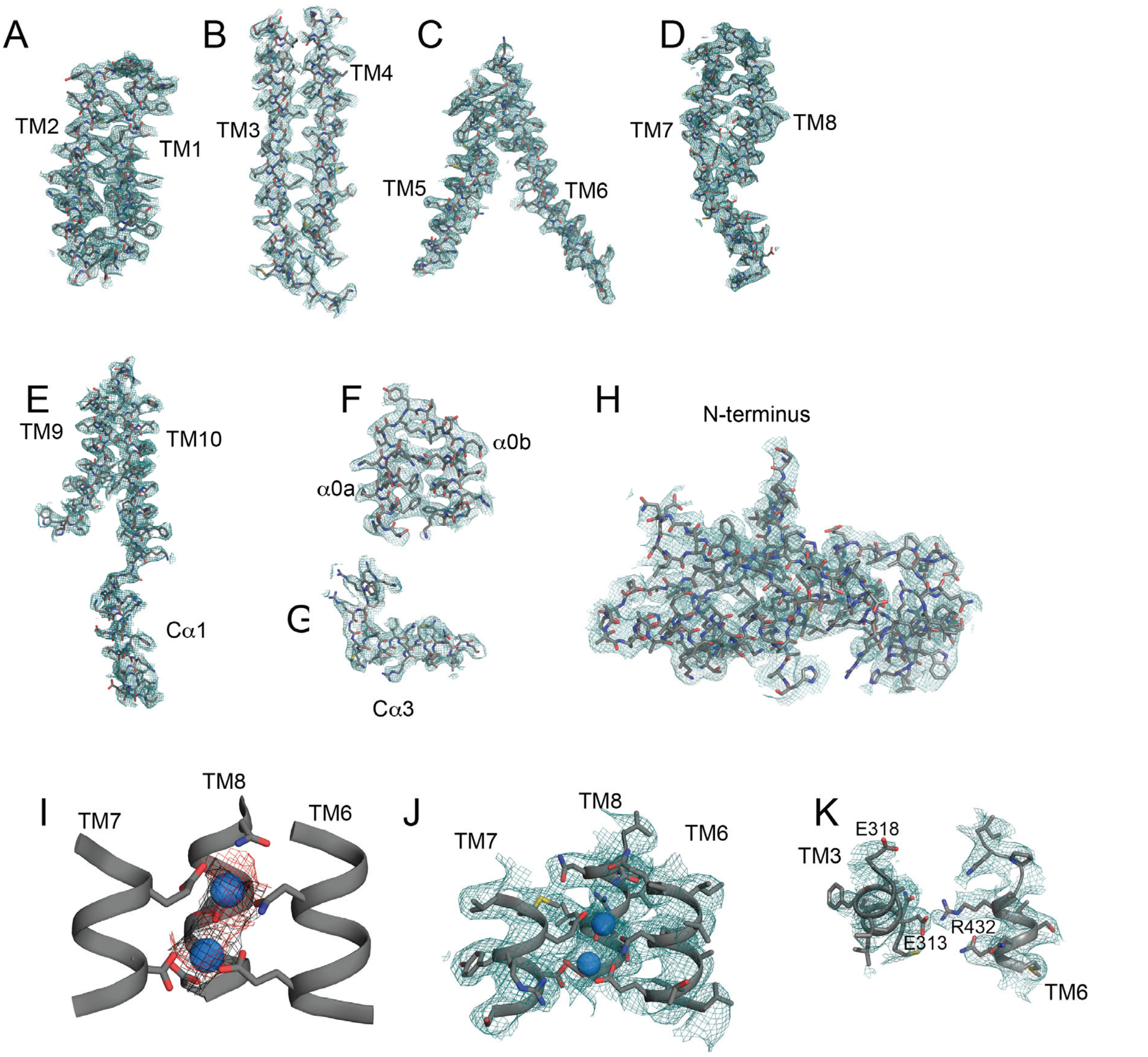

Supplementary Figure 14. Representative cryo-EM density for nhTMEM16 L302A. (A-H), The cryo-EM density (teal mesh) is overlaid with the atomic model (grey sticks). Heteroatoms are colored as follows: oxygen is red, nitrogen is blue, and sulfur is yellow. (A-E), transmembrane domains TM1-10 and cytosolic α7; (F) Close up of the cytosolic helices α0a-b. (G) Close up of the domain-swapped region Cα3. (H) N-terminus containing Nα1-3 and Nβ1-4. (I) Placement of Ca^{2+} ions (blue) inside EM density map (black) and omit difference map (red). Ca^{2+}-coordinating side chains are shown as sticks. (J) Ca^{2+} binding site consisting of portions of TM6-8 with Ca^{2+} ions shown in blue. (K) The extracellular gate formed by residues E313, E318 (on TM3) and R432 on (TM6).

Supplementary Figure 15

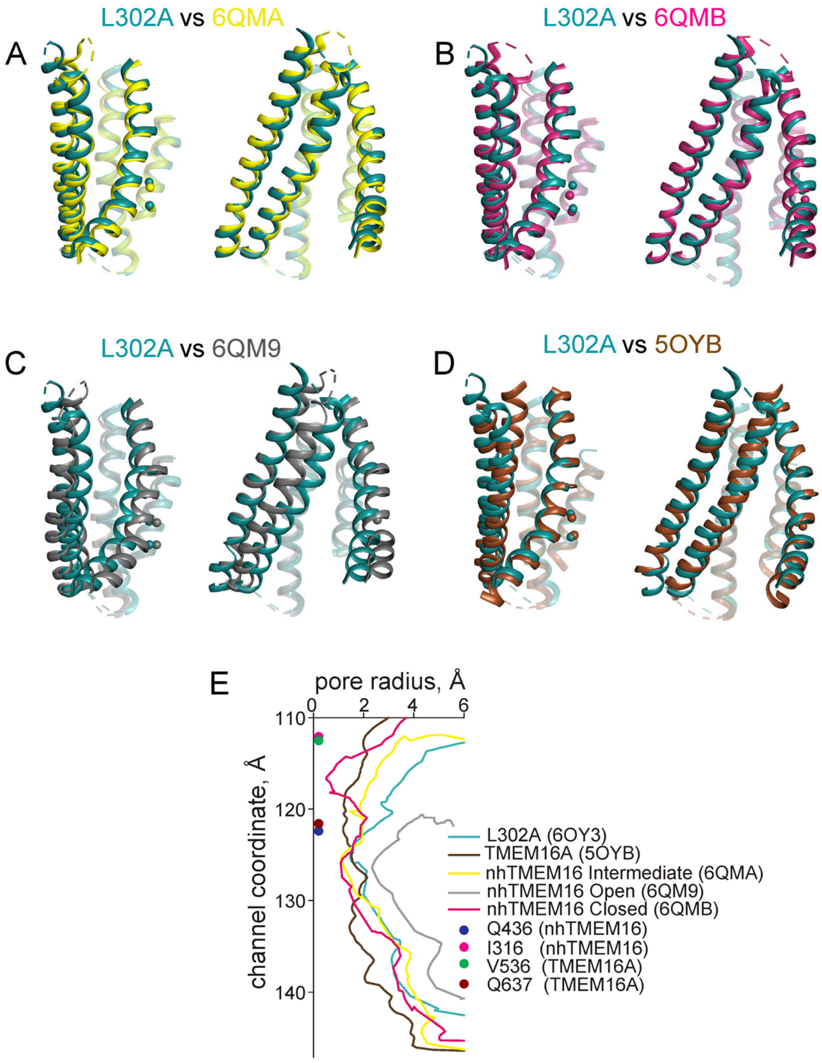

Supplementary Figure 15. Comparison of the groove region structure and pore radii of TMEM16 channels and scramblases. (A-D) Structural alignment of the groove region in the L302A nhMEM16 cryo-EM structure (PDBID 6OY3, teal) with that in (A) WT nhTMEM16 intermediate (PDBID 6QMA; Ca^{2+}-bound nanodiscs, yellow), (B) WT nhTMEM16 Closed (PDBID 6QMB; Ca^{2+}-bound nanodiscs, pink), (C) WT nhTMEM16 Open (PDBID 6QM9; Ca^{2+}-bound structure in nanodiscs, gray), and mTMEM16A (PDBID 5OYB, brown) structures. In these snapshots TMs 3-4 are shown as opaque whereas TMs 5-6 are transparent. The two images in each panel are related to each other by ~90° rotation around the axis of the pore. (E) The radius of the permeation pathway of nhTMEM16 and mTMEM16A was estimated using the program HOLE [5]. L302A (PDBID 6OY3): Teal; WT nhTMEM16 Open (PDBID 6QM9; Ca^{2+}-bound structure in nanodiscs): Gray; Closed (PDBID 6QMB; Ca^{2+}-bound nanodiscs): Pink; Intermediate (PDBID 6QMA; Ca^{2+}-bound nanodiscs): Yellow; Ca^{2+}-bound mTMEM16A (PDBID 5OYB): Brown. Solid circles indicate the position of reference residues in nhTMEM16 (Q436 and I316) or TMEM16A (V536 and Q637) along the z-axis.

Supplementary Figure 16

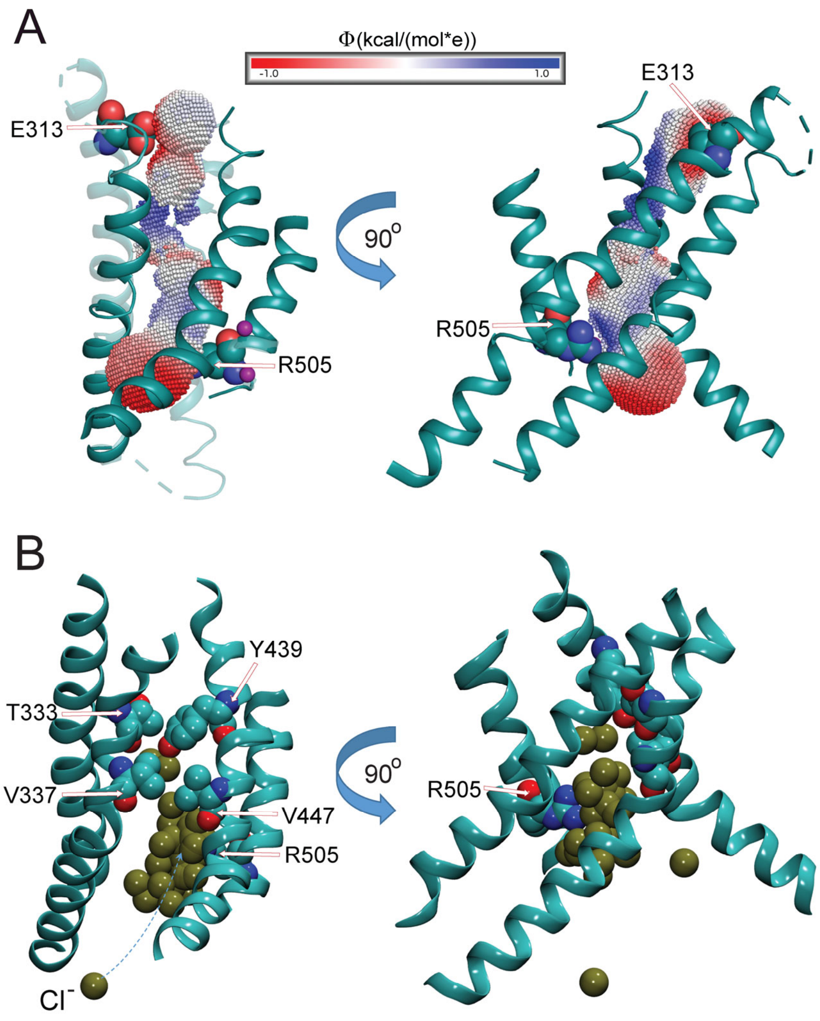

Supplementary Figure 16: The partially closed state of nhTMEM16 protein permits penetration and partial translocation of the Cl^- ions through the groove. (A) Two snapshots related by 90° angle rotation along the membrane normal axis of the cryo-EM structure of nhTMEM16 L302A showing the electrostatic potential (Φ) in the pore region. The electrostatic potential, obtained by solving linear Poisson-Boltzmann equation (see Methods) is depicted on the surface created by the [-1.0; 1.0] kcal/(mol e) range of values in the groove (see also Figure 9 in the main text). The groove helices shown are TMs 3, 4, 6, and 7. Residues E313 and R505 are shown in space fill and labeled. (B) Two snapshots related by 90° angle rotation along the membrane normal axis of the nhTMEM16 protein in the intermediate state, taken from the 15 μs-long WT6 MD simulation (Supplementary Table 1). TMs 3, 4, 6, and 7 are in cartoon representation, and relevant residues are drawn in van der Walls representation and labeled. The path of the penetrating Cl^- ion is shown as the collection of dark green spheres positioned at locations visited by the ion throughout the groove in the first 5 μs of the trajectory.

Supplementary Table 1: Listing of the atomistic MD simulations of nhTMEM16.

nhTMEM16 CONSTRUCT	NUMBER OF LIPIDS	LIPID COMPOSITION	NUMBER OF REPLICATES	TOTAL SIMULATION TIME (μs)	FLIPPING EVENTS	SUBUNITS IN THE OCCLUDED STATE
[a]WT (WT1-2)*	1500	3:1 PE/PG	2	7	0	1
[a]WT (WT3-4)	1500	POPC	2	2	0	1
[a]WT (WT5)	680	3:1 PE/PG	1	10	0	1
[a]WT (WT6)	680	POPC	1	15	0	2
[b]$WT^{ensemble}$ *	680	POPC	24	5	7 (IC to EC)	2
[a]A385W*	1500	3:1 PE/PG	1	2	1 (EC to IC)	0
[a]L302W	1500	3:1 PE/PG	2	7	0	0
[a]L302A	1500	3:1 PE/PG	2	3	0	3

Simulations carried out on Anton2 are identified by the superscript *a*; ensemble simulations carried out with ACEMD are identified by the superscript *b* (see Methods for more details). Simulations marked by "*" are from our previous studies described in [1]. Simulation details include protein construct name, size of the lipid membrane (as the number of lipids), lipid composition of the membranes. For each construct, the columns specify the number of independent replicates, simulation time, the number and direction of lipid flipping events (i.e. from intracellular to extracellular, IC to EC, or vice-versa), and the number of subunits adopting an occluded-groove configuration (see main text).

Supplementary Table 2: Listing of the residue pairs used for dimensionality reduction analysis with the tICA approach

DYNAMIC VARIABLES USED FOR tICA ANALYSIS
T333-Y439
E313-R432
E318-R432
Y439-R432
V337-V447

Supplementary Table 3: Pairwise root-mean-square deviations (RMSD) between the backbone atoms of various structural models of nhTMEM16.

	4WIS	L302A	6QM4	6QM5	6QM6	6QM9	6QMA	6QMB	WT_inter
4WIS	0	2.8	3.1	0.6	0.6	0.7	2.9	3.2	2.4
L302A		0	1.7	2.7	2.8	2.6	1.3	1.6	2.0
6QM4			0	3.0	3.0	2.8	1.0	0.8	2.4
6QM5				0	0.4	0.5	2.8	3.1	2.4
6QM6					0	0.5	2.8	3.1	2.5
6QM9						0	2.6	2.9	2.3
6QMA							0	0.6	2.1
6QMB								0	2.4
WT_inter									0

"L302A" is the cryo-EM structure of the L302A mutant reported in the current study. "WT_inter" is the Ca^{2+}-bound intermediate state of the wild type nhTMEM16 reported in the current study (the last frame of the 15 μs molecular dynamics trajectory of the wild type nhTMEM16 in POPC membrane (Supplementary Table 1) was used for the RMSD calculation). The other entries correspond to the wild type nhTMEM16 protein and are identified by their PDB IDs:

4WIS: Ca^{2+}-bound in detergent (open state) [6]
6QM4: Ca^{2+}-free in nanodiscs[7]
6QM5: Ca^{2+}-bound in detergent[7]
6QM6: Ca^{2+}-free in detergent[7]
6QM9: Ca^{2+}-bound nanodisc (open state)[7]
6QMA: Ca^{2+}-bound nanodisc (intermediate state)[7]
6QMB: Ca^{2+}-bound nanodisc (closed state)[7]

Supplementary Table 4: Statistics of cryo-EM data collection and model refinement for nhTMEM16 L302A/nanodisc complex in the presence of 0.5 mM Ca^{2+}

Data Collection and Processing

Microscope	FEI titan Krios
Camera	Gatan K2 Summit
Nominal Magnification	205000x
Voltage (kV)	300
Exposure time frame/total (s)	0.2/10
Number of Frames	50
Electron exposure total (e^-/Å^2)	70.70
Nominal Defocus range (µm)	-1.5 to -2.5
Pixel Size (Å)	1.0961
Symmetry imposed	C2
Initial/Final micrographs (no.)	4,710/2,696
Initial Particle Images (no.)	1,024,244
Final Particle Images (no.)	47,243
Map resolution (Å) (0.143 FSC)	4.00
Map Resolution Range (Å)	2.9-6
**Refinement**	
Initial model used (PDB code)	4WIS and 6QM5
Model Resolution (Å) (0.5 FSC)	4.20
Map Sharpening B factor (Å^2)	-148.324
**Model Composition**	
Non-hydrogen Atoms	9538
Protein residues	1188
Ligands	4
**R.m.s. deviations**	
Bond length (A)	0.009
Bond angles (°)	1.094
**Validation**	
MolProbity score	1.95
Clashscore	9.28
Poor Rotomers (%)	0.4
EMRinger score	2.00
**Ramachandran Plot**	
Favored (%)	92.88
Allowed (%)	6.94
Disallowed (%)	0.17

REFERENCES

1 Lee, B. C. *et al.* Gating mechanism of the extracellular entry to the lipid pathway in a TMEM16 scramblase. *Nature communications* **9**, 3251 (2018).

2 Bethel, N. P. & Grabe, M. Atomistic insight into lipid translocation by a TMEM16 scramblase. *Proc Natl Acad Sci U S A* **113**, 14049-14054, doi:10.1073/pnas.1607574113 (2016).

3 Cardone, G., Heymann, J. B. & Steven, A. C. One number does not fit all: Mapping local variations in resolution in cryo-EM reconstructions. *Journal of Structural Biology* **184**, 226-236, doi:10.1016/j.jsb.2013.08.002 (2013).

4 Heymann, J. B. Bsoft: Image and Molecular Processing in Electron Microscopy. *Journal of Structural Biology* **133**, 156-169, doi:https://doi.org/10.1006/jsbi.2001.4339 (2001).

5 Smart, O. S., Neduvelil, J. G., Wang, X., Wallace, B. A. & Sansom, M. S. HOLE: a program for the analysis of the pore dimensions of ion channel structural models. *J Mol Graph* **14**, 354-360 (1996).

6 Brunner, J. D., Lim, N. K., Schenck, S., Duerst, A. & Dutzler, R. X-ray structure of a calcium-activated TMEM16 lipid scramblase. *Nature* **516**, 207-212, doi:10.1038/nature13984 (2014).

7 Kalienkova, V. *et al.* Stepwise activation mechanism of the scramblase nhTMEM16 revealed by cryo-EM. *eLife* **8**, doi:10.7554/eLife.44364 (2019).
