# Supplementary material for: Dynamic modulation of the lipid translocation groove generates a conductive ion channel in Ca2+-bound nhTMEM16
Source: Nat Commun. 2019 Oct 31;10:4972. doi: 10.1038/s41467-019-12865-4 (PMC6823365; doi:10.1038/s41467-019-12865-4)
Supplement: Supplementary file 4 — Description of Additional Supplementary Files [file 41467_2019_12865_MOESM4_ESM.pdf]

Title: Supplementary Movie 1

Description: Molecular dynamics simulation of the groove closure in nhTMEM16. Transmembrane helices 3-7 of the protein are shown as white cartoon. The key residues that engage in interactions upon groove closure, V337 and V447, are shown in space-fill representation, respectively colored blue and red. The penetrating lipid is rendered in licorice. The total length of the trajectory is 15 μs.
